# Association between nickel exposure and body compositions in the United States: a population-based cross-sectional study

**DOI:** 10.1186/s12889-023-16483-0

**Published:** 2023-08-25

**Authors:** Xingyang Su, Zilong Zhang, Shi Qiu, Bin Zeng, Mi Yang, Xinyi Huang, Xiaoli Zou, Lu Yang

**Affiliations:** 1 Department of Urology and Institute of Urology, West China Hospital, Sichuan University, Chengdu, 610041 China; 2 West China School of Public Health and West China Fourth Hospital, Sichuan University, Chengdu, 610041 China

**Keywords:** Pollutant, Urinary nickel, Nickel exposure, Body composition, NHANES

## Abstract

**Background:**

Increasing body fat or decreasing muscle and bone mass were associated with worse health outcomes in the adult population. The effects of nickel exposure on body composition are not known. The aim of the current study was to investigate the relationship between urinary nickel levels and body compositions.

**Materials and methods:**

Two thousand seven hundred sixty-two participants were included in the analysis from the National Health and Nutrition Examination Surveys of 2017–2018 after excluding participants who have missing data on urinary nickel and those with missing all body mass component data. We used weighted generalized linear models to explore the relationship between urinary nickel and body mass components under interpolating missing covariable values. Simultaneously, sensitivity analyses and subgroup analysis were conducted to verify stability of analysis result. Curve fitting and saturation effect analysis were used to explore the possible nonlinear relationship between urine nickel and body compositions.

**Results:**

Among the 2,762 participants, the average urinary nickel level was 1.58 ug/L. The weighted generalized linear models, the sensitivity analyses and subgroup analyses found no significant linear relationship between urinary nickel and body compositions. For body weight, BMI, TLM, ALM, TRF, TOF and BMC, the urine nickel saturation effect values were 0.76, 0.74, 0.5, 0.67, 0.64, 0.48, and 0.45 ug/L, respectively. For each 1 ug/L rise in urinary nickel levels at levels below the turning point, body weight increases (β = 9.06, 95% CI = 2.75, 15.36, *p* = 0.01), BMI increases (β = 3.20, 95% CI = 1.36, 5.05, *p* =  < 0.001), TLM decreases (β = -47.39, 95% CI = -97.38, 2.59, *p* = 0.06), ALM decreases (β = -37.25, 95% CI = -63.25, -11.24, *p* = 0.01), TRF increases (β = 20.68, 95% CI = 1.50, 39.86, *p* = 0.03), TOF increases (β = 57.92, 95% CI = -0.12, 115.95, *p* = 0.05), and BMC decreases (β = -6.84, 95% CI = -12.64, -1.04, *p* = 0.02).

**Conclusions:**

In summary, our study demonstrated that a dose–response relationship exists between urinary nickel and body compositions, with a low inflection point level of urinary nickel for the saturation effect.

**Supplementary Information:**

The online version contains supplementary material available at 10.1186/s12889-023-16483-0.

## Background

Evaluation of body composition is a fundamental part of metabolism and physiology research in humans, due to accurate estimates of the various components of body mass, including body fat, lean body mass, and bone mineral content [1]. Increasing body fat or decreasing muscle and bone mass were associated with worse health outcomes in the adult population [2]. Indicators of body fat mass, such as high visceral fat or normal-weight central obesity, are strongly correlated with cardiovascular disease risk and mortality [3, 4]. Low lean body mass was considered independently linked to insulin resistance, diabetes, metabolic syndrome, and musculoskeletal injuries, and impedes one's functional capacity by reduced resting energy metabolism, fatigue, weakened neuromuscular function, and an heightened risk of injury [1, 5]. Osteoporosis, a major public health issue, imposes a heavy social and economic burden due to its associated fractures, pain, physical impairments, deformities, disabilities, and psychological disorders. Osteoporosis is characterized by a decrease in bone mineral content and density [6]. Gaining insight into the specific changes in body composition can improve our knowledge of obesity, metabolic health, aging, and chronic illnesses, leading to more precise and tailored medical care.

Nickel (Ni), the 28th element on the periodic table, is presented as a variety of minerals in soils, meteorites and volcanic ash in the Earth's crust [7, 8]. Nickel-containing foods are very common, such as vegetables, fruits, grains, cocoa beans, soybeans, peanuts, dark chocolate, seafood [7]. Humans generally do not have deficiency of nickel in their body as it is abundant in nature and in most food that humans consume [9]. The exposure to nickel in humans can also derive from many other sources, which lead to an excess absorption of nickel by the human body. The atmosphere, water, and soil have been exposed to an excessive amount of nickel due to the combustion of fossil fuels, the discharge, or infiltration, of nickel-containing industrial waste [10, 11]. Nickel alloys and nickel compounds are widely used in daily necessities and modern industries, such as inexpensive jewelry, household goods, electrical equipment, coins, food processing, catalysts, and even medical prostheses and orthodontic materials [7, 9, 12]. Furthermore, cigarette smoking is also a major source of nickel exposure, with a maximum of 0.023 mg of nickel per forty cigarettes smoked [8]. Although nickel is an essential mineral in the human body, its immunotoxicity, neurotoxicity, and carcinogenicity are attributed to a variety of health diseases, such as allergic contact dermatitis, pulmonary fibrosis, lung and nasal cancers, asthma, kidney disease, and cardiovascular disease, depending on excessive doses and long exposure to different types of compounds [7, 13]. Lung cancer and nasal cancer have long been identified as occupational diseases of refinery workers caused by long-term occupational exposure to nickel, and a recent study also confirms that relatively low cumulative levels of occupational nickel exposure are associated with an increased risk of lung cancer [14, 15]. Results from a meta-analysis of 28 studies involving patch-tested individuals from the general population showed that 20% of them had contact allergies, with nickel being the most common allergen at 11.4% [16]. Previous studies have found significantly elevated serum nickel levels in patients with acute myocardial infarction or unstable angina, along with a tendency to increase the risk of atherosclerotic plaque, heart failure, and heart attack with increasing exposure [17, 18]. Some studies have provided evidence that exposure to Ni is linked with a decline in kidney function and an increased risk of craniosynostosis [19, 20]. Due to the widespread use of nickel alloys and nickel compounds, great concern has been raised about health problems associated with nickel [21], but the relationship between nickel exposure and body mass components has not been reported.

Therefore, the objective of the study is to evaluate the relationship between urinary nickel and body composition among the participants of the National Health and Nutrition Examination Survey (NHANES). Understanding these associations will help to improve our current understanding of how nickel exposure affects body composition, strengthen the emphasis on nickel exposure, and may lead to new recommendations for preventing body composition-related health problems and diseases.

## Methods

### Study design and participants

The data we investigated originated from a publicly accessible database—the NHANES database. The NHANES project began in the early 1960s and focuses on different population groups or health topics in the form of surveys. Since 1999, this survey has become an ongoing program, taking a nationally representative sample survey of around 5,000 people each year, and focusing on the health and nutritional status of the national population to meet emerging health needs [22]. The national cross-sectional survey is conducted by the National Center for Health Statistics, whose protocol was audited and approved by the National Center for Health Statistics Research Ethics Review Board. The questionnaire, physical examination, and laboratory examination were only performed after the participants had signed a written informed consent. More detailed information on survey methods and protocols is available on the official website (http://www.cdc.gov/nchs/nhanes.htm).

**Fig. 1:**
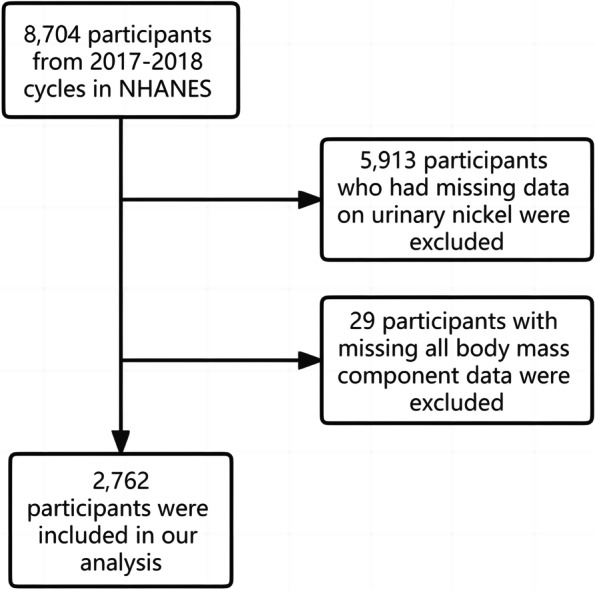
Flow chart of inclusion and exclusion criteria for our analysis

The study included 8,704 individuals from the NHANES cycle from 2017 to 2018 because only the survey of 2017–2018 examined urinary nickel concentrations. After excluding participants who had missing data on urinary nickel (*n* = 5913) and participants with missing all body mass component data (*n* = 29), 2762 participants were included in the final analysis. Body mass components were outcomes in this study, including weight, body mass index (BMI), total lean mass (TLM), appendicular lean mass (ALM), bone mineral content (BMC), total fat (TOF), and trunk fat (TRF). Figure 1 depicts the selection process.

### Urinary nickel

The main routes of nickel excretion are urine and feces, while urine is a reliable specimen for nickel exposure detection [23, 24]. As a very sensitive laboratory technology to measure multiple elements at low concentrations, inductively coupled plasma‒mass spectrometry (ICP‒MS) was used for the quantification of urinary nickel concentrations [25]. There is no time requirement for collecting urine samples, and no fasting or special diet is required prior to urine collection. Urine samples were collected, freeze-transported and finally stored at -20 °C until analysis (short-term storage for 2 weeks at 2–8 °C is acceptable). The principle of ICP‒MS is summarized as follows: First, urine samples were atomized in a high-temperature plasma generated by flowing argon via RF power coupling and then ionizes the atoms. Second, after entering the mass spectrometer, the ions, along with argon, pass through a focused region (the Universal unit), a quadrupole mass filter, and are counted in rapid order on the detector. In this survey, the detection threshold of nickel in urine was 0.31 ug/L [26]. Incomplete data or improbable values were sent to the performing laboratory for confirmation. Specific sample detection and quality control methods are available in the laboratory procedure manual for detecting arsenic, chromium, and nickel in urine by ICP‒MS. (https://wwwn.cdc.gov/nchs/data/nhanes/2017-2018/labmethods/UTAS-J-UCM-J-UNI-J-MET-508.pdf).

### Body mass components

Body measurement data, including weight and BMI, were collected in the Mobile Examination Center by trained health technicians. The dual-energy X-ray absorptiometry (DEXA) scans, as the most widely accepted method of measuring body composition, provides bone and soft tissue measurements for the total body, for both arms and both legs, the trunk, and the head in NHANES [27, 28]. These body mass components including ALM (g), TLM (g), BMC (g), TOF (g) and TRF (g) were measured using DEXA for eligible participants. ALM, a well-recognized proxy for skeletal muscle mass [29], was calculated by summing up the lean mass of the four upper and lower limbs (excluding bone mineral content). All TLM, TOF and TRF can be directly extracted from the examination data of NHANES. BMC was also included in this analysis because of the extensive evidence of constant cross-talk and wasting consistency between the skeletal muscle and bone during aging and pathology [30]. Considering the effect of body mass on these several variables measured by DEXA, the weight-adjusted variables (g per kg of body mass; g/kg BM) converted from the original variables divided by weight were used as the final outcome variables [31].

### Covariates

The most basic demographic information, such as gender, age, race, economic condition, and education level was obtained through questionnaires. Race was classified as non-Hispanic white, non-Hispanic black, Other Hispanic and other Race. The income poverty rate is used to measure economic conditions, with a rate of  ≤ 1.3 being considered low, 1.3–3.5 as medium, and  ≥ 3.5 as high. Education level is classified as less than high school, high school or general educational development, and above high school. To make urinary nickel more accurately reflect the level of nickel exposure in the human body, we included the estimated glomerular filtration rate (eGFR), an indicator of renal function. We used the Chronic Kidney Disease Epidemiology Collaboration (CKD-EPI) creatinine equation, which is more accurate than the Modification of Diet in Renal Disease Study equation, to calculate eGFR [32]. The eGFR of CKD-EPI equation = 141 × min(Scr/κ, 1)α × max(Scr/κ, 1)-1.209 × 0.993age × 1.018 [if female] × 1.159 [if black], where Scr is serum creatinine, κ is 0.7 for females and 0.9 for males, α is -0.329 for females and -0.411 for males, min indicates the minimum of Scr/κ or 1, and max indicates the maximum of Scr/κ or 1. Race variable is not taken into account in the formula in our study as it is no longer recommended [33].

### Statistical analysis

Continuous variables are denoted by the mean (standard deviation), while categorical variables are represented as the number (percentage). NHANES strata information, primary sampling unit, and two-year sampling weights accounted for the complex survey design of NHANES to produce unbiased nationally representative estimates, so a sample weight was assigned to each participant [34]. The Kruskal–Wallis H test was applied for continuous variables, and a weighted chi-square test was applied for categorical variables to complete comparisons between the urinary nickel quartiles. We interpolated the data due to the partial absence of the covariates. Missing values were entered by using multiple imputation with chained equations, which predicts the most likely value for each missing value based on the other covariables’ characteristics of the participant. Firstly, We used weighted generalized linear models to explore the relationship between urinary nickel and body mass components. Weighted generalized linear models included a non-adjusted model, an adjusted-I model (adjusted for age, sex, race and eGFR) and an adjusted-II model (adjusted for age, sex, race and eGFR, the ratio of income-poverty, and education level). Secondly, sensitivity analyses were performed using a weighted generalized linear model after removing the outliers of urinary nickel to validate the stability of the analysis results. The outliers were defined as urinary nickel levels outside the Mean + -3SD range (SD stands for standard deviation). Simultaneously, we confirmed the stability of the results by carrying out subgroup analyses of gender, age, race and eGFR, ratio of income-poverty, and education level. To explore the possible nonlinear relationship and inflection point between urine nickel and body composition, smoothed curve fitting and saturation effect analysis were used in our analysis after adjusting for age, sex, race, eGFR, the ratio of income-poverty, and education level. To calculate the inflection point, a recursive algorithm is being utilized to construct a weighted two-piecewise linear regression model. All analyses were performed using EmpowerStats (www. empow ersta ts. com, X&YSolution, Inc.) and the statistical software package R (http://www.Rproject.org, The R Foundation). A two-tailed p value < 0.05 was considered statistically significant.

## Results

### Baseline characteristics of participants

Table 1 shows the baseline characteristics of NHANES participants categorized by their urinary nickel levels between 2017 and 2018. Among the 2,762 participants, the average urinary nickel level was 1.58 μg/L, and the average urinary nickel levels for quartiles 1–4 were 0.44 μg/L, 0.98 μg/L, 1.67 μg/L, and 3.93 μg/L, respectively.There were no significant differences between weight, BMI, ALM, TLM, BMC, and TRF between the four urinary nickel quartiles except for TOF (*p* = 0.08, 0.09, 0.66, 0.15, 0.61, 0.54, and 0.04, respectively). Compared with Q1, Q2, and Q3 of urinary nickel, participants in Q4 were more likely to be younger, of poorer economic condition, and of other Hispanic or other races, while eGFR, male–female ratio, and education level made no difference among the four groups.

**Table 1:** Baseline characteristics of NHANES participants by categories of urinary nickel levels between 2017 and 2018 (*n* = 2762)

Characteristic	Total	Q1	Q2	Q3	Q4	*P*-value
Urinary nickel, ug/L, (N) mean (SD)	(2762) 1.58 (0.05)	(684) 0.44 (0.01)	(697) 0.98 (0.01)	(687) 1.67 (0.01)	(694) 3.93 (0.19)	< 0.01
Weight, kg, (N) mean (SD)	(2756) 76.60 (0.62)	(684) 76.95 (1.35)	(694) 80.37 (1.81)	(686) 75.57 (1.43)	(692) 72.20 (1.80)	0.08
BMI, kg/m2, (N) mean (SD)	(2753) 27.95 (0.21)	(684) 27.78 (0.43)	(693) 28.80 (0.57)	(684) 27.87 (0.43)	(692) 27.13 (0.49)	0.09
TLM, g/kg BM, (N) mean (SD)	(1199) 651.38 (4.10)	(318) 642.40 (5.64)	(307) 654.74 (11.96)	(289) 660.21 (8.41)	(285) 650.31 (5.57)	0.15
ALM, g/kg BM, (N) mean (SD)	(1320) 264.38 (3.50)	(348) 262.06 (5.09)	(337) 263.06 (7.98)	(329) 262.83 (7.80)	(306) 272.55 (5.29)	0.66
TRF, g/kg BM, (N) mean (SD)	(1244) 146.49 (2.09)	(328) 150.64 (3.59)	(320) 145.80 (5.79)	(305) 143.65 (4.06)	(291) 144.34 (3.00)	0.54
TOF, g/kg BM, (N) mean (SD)	(1151) 322.84 (4.51)	(308) 333.56 (5.83)	(296) 318.48 (12.28)	(275) 311.35 (8.48)	(272) 325.63 (5.55)	0.04
BMC, g/kg BM, (N) mean (SD)	(1151) 30.50 (0.32)	(306) 30.44 (0.39)	(295) 30.28 (0.63)	(277) 31.15 (0.68)	(273) 30.06 (0.52)	0.61
Age, years, (N) mean (SD)	(2762) 39.87 (0.94)	(684) 41.73 (1.48)	(697) 40.73 (1.37)	(687) 39.61 (1.37)	(694) 36.38 (1.44)	0.04
eGFR, ml/(min* 1.73m^2^), (N) mean (SD)	(1907) 99.70 (25.91)	(512) 98.57 (22.68)	(522) 100.97 (25.92)	(458) 98.28 (26.20)	(415) 101.50 (29.13)	0.38
Gender, (N) %						0.63
Male	(1362) 49.38	(305) 46.33	(352) 52.09	(356) 51.11	(349) 47.90	
Female	50.62	(379) 53.67	(345) 47.91	(331) 48.89	(345) 52.10	
Race, (N) %						0.01
Other Hispanic	(430) 12.29	(119) 12.68	(97) 11.46	(103) 11.34	(111) 14.10	
Non-Hispanic White	(235) 7.85	(65) 8.57	(60) 6.72	(64) 8.91	(46) 7.15	
Non-Hispanic Black	(897) 66.57	(217) 69.33	(241) 68.88	(218) 65.09	(221) 61.15	
Other Race	(634) 13.29	(119) 9.43	(173) 12.94	(169) 14.66	(173) 17.60	
Ratio of income-poverty, (N) %						0.02
Low	(819) 24.86	(176) 19.05	(214) 24.12	(207) 28.07	(222) 30.32	
Middle	(948) 35.24	(238) 35.73	(240) 34.07	(234) 34.04	(236) 37.48	
High	(657) 39.90	(188) 45.22	(166) 41.80	(153) 37.90	(150) 32.20	
Education level, (N) %						0.55
Less than high school	(138) 4.95	(39) 5.21	(31) 3.99	(34) 4.44	(34) 6.80	
High school or general educational development	(198) 11.35	(41) 8.89	(54) 10.86	(57) 12.90	(46) 14.30	
Above high school	(955) 83.70	(271) 85.90	(264) 85.15	(223) 82.66	(197) 78.89	

*BMI* body mass index, *TLM* total lean mass, *ALM* appendicular lean mass, *BMC* bone mineral content, *TOF* total fat, *TRF* trunk fat, *eGFR* estimated glomerular filtration rate, *Q* quartiles

### The association between nickel exposure and body compositions

The association between urinary nickel and body components, calculated using weighted generalized linear models, including the non-adjusted model, adjusted-I model, and adjusted-II model, is presented in Table 2. After data imputation, we also didn't observe a significant association between the body compositions, including the weight, BMI, TLM, ALM, TRF, TOF, and BMC, and urinary nickel in the adjusted-II model (*p* = 0.20, 0.54, 0.20, 0.22, 0.22, 0.14, and 0.07, respectively). Table 3 presents the results of the sensitivity analyses after removing the extreme values of urinary nickel. There was a negative association between BMC and urinary nickel (β = -0.42, 95% CI = -0.88 to -0.05) with a *p*-value of 0.05. Similarly, body compositions, including weight, BMI, TLM, ALM, TRF, and TOF were not found to be related to nickel urine levels in the absence of extreme values (*p* = 0.29, 0.79, 0.37, 0.82, 0.31, and 0.27, respectively). Subgroup analyses of sex, age, race, eGFR, ratio of income to poverty, and education level also did not find a significant interaction, as shown in Tables S1-S6. The above analyses found no significant linear relationship between urinary nickel and body compositions.

**Table 2:** Association between nickel exposure and body mass components among NHANES participants in 2017—2018 after multiple imputation

outcomes	N	Non-adjusted model		Adjusted-I model		Adjusted-II model	
		β (95% CI)	*P*-value	β (95% CI)	*P*-value	β (95% CI)	*P*-value
Weight, kg	2756	-1.11 (-1.96, -0.26)	0.02	-0.49 (-1.05, 0.07)	0.12	-0.45 (-1.38, 0.48)	0.20
BMI, kg/m2	2753	-0.20 (-0.42, 0.02)	0.10	-0.06 (-0.23, 0.10)	0.49	-0.06 (-0.34, 0.22)	0.54
TLM, g/kg BM	1199	-0.19 (-2.58, 2.19)	0.88	-0.99 (-2.23, 0.25)	0.16	-1.01 (-3.13, 1.11)	0.20
ALM, g/kg BM	1320	-0.59 (-3.05, 1.87)	0.65	-1.30 (-2.97, 0.36)	0.16	-1.20 (-3.84, 1.44)	0.22
TRF, g/kg BM	1244	-0.30 (-2.01, 1.40)	0.73	0.62 (-0.17, 1.41)	0.16	0.59 (-0.21, 1.38)	0.22
TOF, g/kg BM	1151	0.39 (-1.99, 2.76)	0.75	1.31 (-0.14, 2.75)	0.11	1.35 (-1.01, 3.72)	0.14
BMC, g/kg BM	1151	-0.10 (-0.21, 0.01)	0.09	-0.16 (-0.33, 0.01)	0.05	-0.17 (-0.38, 0.05)	0.07

*BMI* body mass index, *TLM* total lean mass, *ALM* appendicular lean mass, *BMC* bone mineral content, *TOF* total fat, *TRF* trunk fat

Non-adjusted model adjust for: None; Adjusted-I model adjust for: age, gender, race, and eGFR; Adjusted-II model adjust for: age, gender, race, eGFR, the ratio of income-poverty, and education level

**Table 3:** Sensitivity analyses for association between nickel exposure and body mass components without extreme values of urinary nickel

outcomes	N	Non-adjusted model		Adjusted-I model		Adjusted-II model	
		β (95% CI)	*P-*value	β (95% CI)	*P*-value	β (95% CI)	*P*-value
Weight, kg	2723	-1.87 (-2.77, -0.98)	< 0.01	-0.82 (-1.94, 0.29)	0.19	-0.69 (-2.48, 1.11)	0.29
BMI, kg/m2	2720	-0.47 (-0.70, -0.23)	< 0.01	-0.07 (-0.39, 0.26)	0.70	-0.05 (-0.59, 0.49)	0.79
TLM, g/kg BM	1186	3.88 (0.16, 7.61)	0.04	-2.04 (-6.10, 2.03)	0.35	-2.21 (-9.20, 4.78)	0.37
ALM, g/kg BM	1305	1.61 (-1.18, 4.40)	0.26	0.28 (-3.02, 3.57)	0.87	0.39 (-4.82, 5.61)	0.82
TRF, g/kg BM	1229	-3.42 (-5.63, -1.21)	< 0.01	1.51 (-0.96, 3.97)	0.27	1.53 (-2.68, 5.73)	0.31
TOF, g/kg BM	1139	-3.45 (-7.43, 0.52)	0.09	2.67 (-1.59, 6.93)	0.25	2.92 (-4.33, 10.17)	0.27
BMC, g/kg BM	1139	-0.06 (-0.34, 0.22)	0.68	-0.40 (-0.69, -0.11)	0.03	-0.42 (-0.88, 0.05)	0.05

*BMI* body mass index, *TLM* total lean mass, *ALM* appendicular lean mass, *BMC* bone mineral content, *TOF* total fat, *TRF* trunk fat

Non-adjusted model adjust for: None; Adjusted-I model adjust for: age, gender, race, and eGFR; Adjusted-II model adjust for: age, gender, race, eGFR, the ratio of income-poverty, and education level

### The non-linearity and saturation effect analysis between urinary nickel and body compositions

**Fig. 2:**
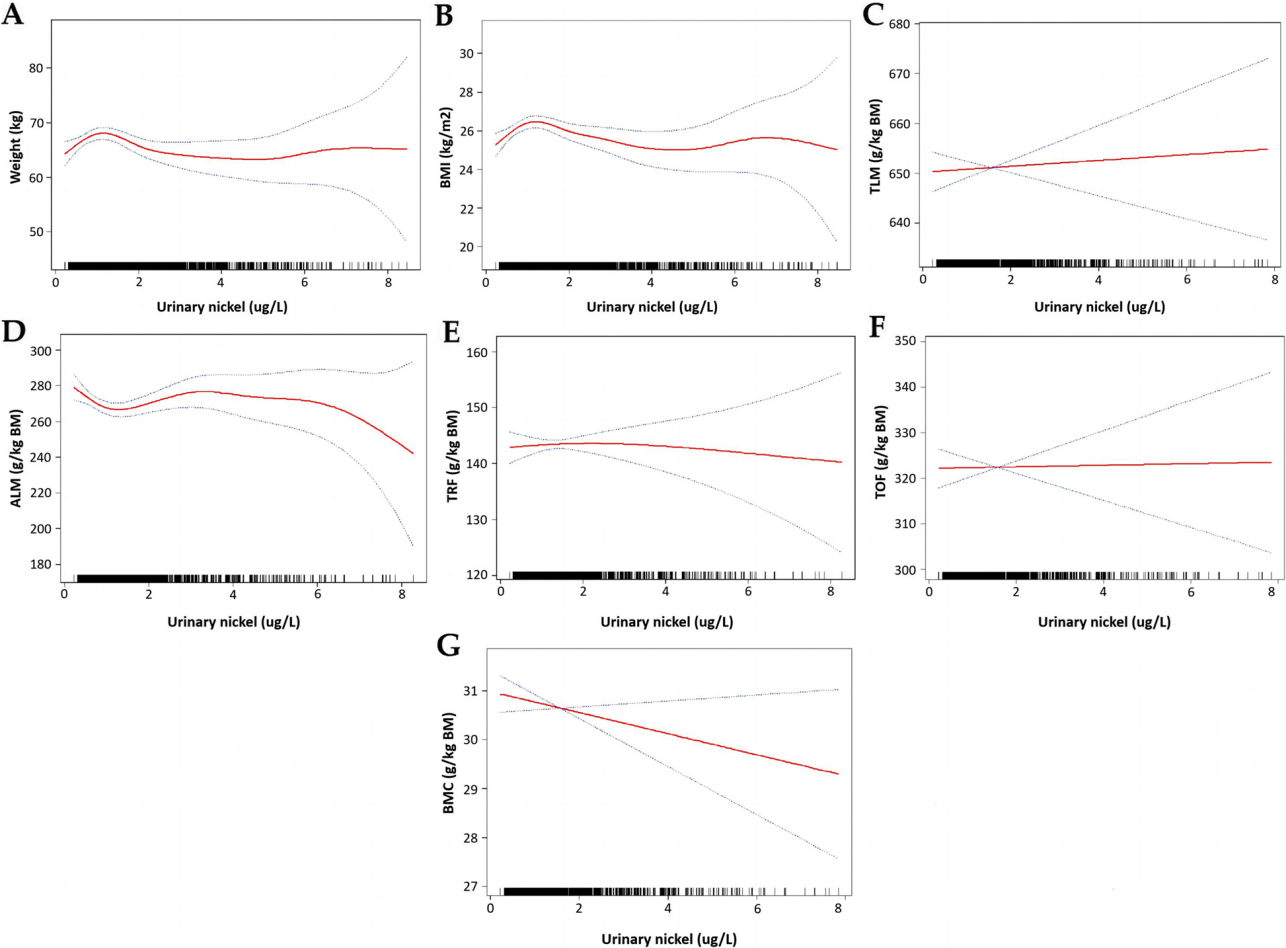
The non-liner association between urinary nickel and body compositions. The solid red line represents the smooth curve fit between variables. Blue bands represent the 95% confidence interval from the fit. Age, sex, race, eGFR, the ratio of income-poverty, and education level were adjusted. BMI: body mass index; TLM: total lean mass; ALM: appendicular lean mass; BMC: bone mineral content; TOF: total fat; TRF: trunk fat

The non-linear relationship between urinary nickel and body compositions were characterized by smoothed curve fittings (Fig. 2). We observed nonlinear relationships between urinary nickel and body compositions with significant inverted U-shaped curves between urinary nickel and weight, BMI, and TRF, and a U-shaped curve between urinary nickel and ALM. There was a noticeable saturation effect between urine nickel level and body compositions (Table 4). For body weight, BMI, TLM, ALM, TRF, TOF, and BMC, the urine nickel saturation effect values were 0.76, 0.74, 0.50, 0.67, 0.64, 0.48, and 0.45 μg/L, respectively. Though the log-likelihood ratio of the saturation effect analysis of TLM and urine nickel was 0.06, the effect values vary widely. The effect value was -47.29 when the urinary nickel level was below 0.5 μg/L and the effect value became 1.75 when the urinary nickel level exceeded 0.5 μg/L. For each 1 μg/L rise in urinary nickel levels at levels below the turning point, body weight increases by 9.06 kg, BMI increases by 3.20 kg/m2, TLM decreases by 47.39 g/kg BM, ALM decreases by 37.25 g/kg BM, TRF increases by 20.68 g/kg BM, TOF increases by 57.92 g/kg BM, and BMC decreases by 6.84 g/kg BM (*p* < 0.01, < 0.001, 0.06, 0.01, 0.03, 0.05, and 0.02, respectively). At levels above saturation effect values, urinary nickel levels were not associated with TLM, ALM, TRF, TOF, and BMC, but body weight and BMI were inversely associated with the urinary nickel level (*p* < 0.01 and < 0.01, respectively).

**Table 4:** The saturation effect analyses of urinary nickel on body mass components

Outcome	The saturation effect analysis			
	Turning point (K), ug/L	< K, effect 1, β(95%CI) *P*-value	> K, effect 2, β (95%CI) *P*-value	Log-likelihood ratio
Weight, kg	0.76	9.06 (2.75, 15.36) < 0.01	-1.21 (-2.03, -0.40) < 0.01	< 0.01
BMI, kg/m2	0.74	3.20 (1.36, 5.05) < 0.001	-0.34 (-0.57, -0.12) < 0.01	< 0.001
TLM, g/kg BM	0.5	-47.39 (-97.38, 2.59) 0.06	1.75 (-1.40, 4.89) 0.28	0.06
ALM, g/kg BM	0.67	-37.25 (-63.25, -11.24) 0.01	1.41 (-1.44, 4.26) 0.33	0.01
TRF, g/kg BM	0.64	20.68 (1.50, 39.86) 0.03	-1.04 (-3.04, 0.96) 0.31	0.03
TOF, g/kg BM	0.48	57.92 (-0.12, 115.95) 0.05	-1.08 (-4.47, 2.32) 0.53	0.05
BMC, g/kg BM	0.45	-6.84 (-12.64, -1.04) 0.02	-0.10 (-0.39, 0.20) 0.53	0.02

*BMI* body mass index, *TLM* total lean mass, *ALM* appendicular lean mass, *BMC* bone mineral content, *TOF* total fat, *TRF* trunk fat, *K* turning point

Age, sex, race, eGFR, the ratio of income-poverty, and education level were adjusted

## Discussion

In this U.S. population-based cross-sectional study, a significant non-linear relationship between urinary nickel and body compositions was observed. When the urinary nickel level was lower than the saturation effect values, body weight, BMI, TRF, and TOF were positively associated with the level of urinary nickel, whereas TLM, ALM, and BMC had a negative association with the level of urinary nickel. Changes in TLM, ALM, TRF, TOF, and BMC showed a saturation effect at very low urinary nickel levels (0.4–0.7 μg/L). However, body weight and BMI were inversely associated with the urinary nickel levels above the value of the saturation effect.

The relationship between urinary nickel and weight, BMI, human muscle mass fat mass was significant only in very low nickel exposure. Nickel is an essential component of many enzymes in the human body, such as s-methyl coenzyme-M (CoM) reductase, acetyl-CoA synthase, CO dehydrogenase, Ni-superoxide dismutase, glyoxalase I, and cis–trans isomerase [9], which are involved in the metabolism of lipids, carbohydrates and amino acids and the absorption of iron. When urinary nickel level was lower than saturation effect values, body weight, BMI, TRF and TOF were positively associated with the level of urinary nickel. Previous studies reported that nickel exposure in animals could cause weight loss, blood glucose and lipid alterations, and fatty infiltration of the liver [35–37]. The implantation of nickel pellets into mouse muscle resulted in the downregulation of energy metabolism pathways, which affected mitochondrial and lysosome functions in cells [38]. Oxidative stress is one of the mechanisms of nickel toxicity, which can disrupt the balance of glutathione reductase and the mitochondrial antioxidant defense system through the formation of nickel-mercaptan complexes [39, 40]. The overproduction of free radicals and reactive oxygen species through the Fenton reaction and direct electron transfer leads to cell death and reduces the number of viable cells [39, 41, 42]. A large body of evidence suggests that metabolic disorders caused by the molecular mechanisms of oxidative stress and inflammation play an important role in obesity, a disease accompanied by low-grade chronic inflammation [43–46]. At levels above saturation effect values, body weight and BMI were inversely associated with urinary nickel level. Resembling the results in the our study, previous studies have shown that even low doses of nickel can have deleterious health effects on carotid atherosclerosis that are eliminated at high levels of exposure [47].

When urinary nickel level was lower than saturation effect values, TLM, ALM and BMC had a negative association with the level of urinary nickel. In our study, it is logical to observe skeletal muscle and bone loss based on the coupling of them [30]. Muscle and bone interact with each other on both a molecular and mechanical level. Studies have revealed that a lack of lean weight can detrimentally influence BMC and a greater skeletal muscle mass is correlated with a higher BMC for adolescents [1, 48]. Sarcopenia is a loss of muscle mass and function in which skeletal muscle inflammation is the main mechanism [49]. The implantation of nickel or nickel-based alloys has been shown to cause inflammatory infiltration of macrophages and lymphocytes in connective tissue and muscle, possibly due to its promotion of intercellular cell adhesion molecule-1 expression, reinforcing its role in the recruitment of inflammatory cells [50]. Inflammatory cytokines represented by C-reactive proteins have been shown to stimulate protein catabolism and inhibit muscle synthesis, leading to muscle atrophy [51]. Our study confirmed a negative association between urinary nickel and BMC. Previous studies confirmed that there was no difference in the mass histological characteristics of rabbit femurs between the test group supplemented with nickel chloride and the control group [52], and bone implants of the Ni–Ti material do not impair the osteotomy healing response, mineralization, or remodelling in rats [53]. However, there are changes in calcium metabolism and bone structure and composition in the case of nickel deprivation [54], which indicates that nickel affects the mineralization and formation mechanism of bone. S. Morais et al. found that the metal ion Ni in vitro cell experiments in osteoblast-like cell cultures decreased and delayed tissue mineralization ability by changing the level and temporal expression of alkaline phosphatase, which had a significant effect on the osteoblast phenotype [55]. Arihiko Kanaji et al. also demonstrated that high concentrations of Ni ions had significant cytotoxic effects on murine long bone-derived osteocytes, which can cause osteocyte death [56]. Our study found an effect of nickel exposure on the mineral composition of human bone, but evidence from in vivo experimental models of the nickel effect on bone tissue is still lacking [57].

To our knowledge, our study is the first to examine the association between urine nickel and body compositions in a population and found that very small amounts of nickel exposure are associated with changes in body compositions, which are associated with worse health outcomes. The present study has also some inevitable shortcomings and limitations. First, the limitations of the cross-sectional survey of NHANES prevented us from determining the causality between urinary nickel and body composition. Second, single urine nickel measurements do not very accurately reflect long-term exposure. Using blood nickel and urina sanguinis or longer urine collection may better reflect nickel exposure, but we were unable to achieve this within the limitations of the method used to measure human nickel levels in NHANES. Third, due to the relatively small number of participants in the 2017–2018 cycle, we were only able to adjust for a few relatively complete covariables, such as age, sex, race, eGFR, ratio of income-poverty, and education level after using multiple imputation with chained equations.

## Conclusion

In summary, our study demonstrated that a dose–response relationship exists between urinary nickel and body compositions, with a low inflection point level of urinary nickel for the saturation effect. To better Identify the effect of nickel exposure on human body compositions, further prospective studies and more in-depth exploration of mechanisms are needed.

### Supplementary Information

**Additional file 1:** **Table S1. **Subgroup analyses about gender between urinary nickel and body mass components in NHANES 2017–2018. **Table S2.  **Subgroup analyses about age between urinary nickel and body mass components in NHANES 2017–2018. **Table S3. **Subgroup analyses about race between urinary nickel and body mass components in NHANES 2017–2018. **Table S4. **Subgroup analyses about eGFR between urinary nickel and body mass components in NHANES 2017–2018. **Table S5.** Subgroup analyses about ratio of income-poverty between urinary nickel and body mass components in NHANES 2017–2018. **Table S6. **Subgroup analyses about education level between urinary nickel and body mass components in NHANES 2017–2018.

## Data Availability

The datasets analyzed for this study can be found in the National Health and Nutrition Examination Surveys [https://wwwn.cdc.gov/nchs/nhanes/default.aspx].

## Abbreviations

ALM: Appendicular lean mass
BMC: Bone mineral content
BMI: Body mass index
CKD-EPI: Chronic kidney disease epidemiology collaboration
DEXA: Dual-energy X-ray absorptiometry
eGFR: Estimated glomerular filtration rate
NHANES: National health and nutrition examination surveys
Ni: Nickel
TLM: Total lean mass
TOF: Total fat
TRF: Trunk fat
