# Supplementary material for: Association between nickel exposure and body compositions in the United States: a population-based cross-sectional study
Source: BMC Public Health. 2023 Aug 25;23:1632. doi: 10.1186/s12889-023-16483-0 (PMC10463381; doi:10.1186/s12889-023-16483-0)
Supplement: Supplementary file 1 — Additional file 1: Table S1. Subgroup analyses about gender between urinary nickel and body mass components in NHANES 2017–2018. Table S2. Subgroup analyses about age between urinary nickel and body mass components in NHANES 2017–2018. Table S3. Subgroup analyses about race between urinary nickel and body mass components in NHANES 2017–2018. Table S4. Subgroup analyses about eGFR between urinary nickel and body mass components in NHANES 2017–2018. Table S5. Subgroup analyses about ratio of income-poverty between urinary nickel and body mass components in NHANES 2017–2018. Table S6. Subgroup analyses about education level between urinary nickel and body mass components in NHANES 2017–2018. [file 12889_2023_16483_MOESM1_ESM.docx]

**Table S1: Subgroup analyses about gender between urinary nickel and body mass components in NHANES 2017–2018.**

| **Outcomes** | **Gender= male** | **Gender= female** | **P for interaction** |
| --- | --- | --- | --- |
|  | **β (95%CI) P-value** | **β (95%CI) P-value** |  |
| Weight, kg | -1.00 (-2.87, 0.86) 0.37 | -0.37 (-1.77, 1.03) 0.64 | 0.61 |
| BMI, kg/m2 | -0.03 (-0.51, 0.46) 0.92 | -0.07 (-0.54, 0.40) 0.80 | 0.91 |
| TLM, g/kg BM | -4.21 (-10.08, 1.66) 0.25 | 0.06 (-5.41, 5.52) 0.99 | 0.27 |
| ALM, g/kg BM | 2.09 (-4.09, 8.27) 0.55 | -1.44 (-3.61, 0.72) 0.28 | 0.32 |
| TRF, g/kg BM | 0.96 (-2.38, 4.29) 0.61 | 2.17 (-0.78, 5.12) 0.24 | 0.51 |
| TOF, g/kg BM | 4.74 (-1.47, 10.94) 0.23 | 0.73 (-4.61, 6.07) 0.81 | 0.30 |
| BMC, g/kg BM | -0.46 (-0.88, -0.03) 0.13 | -0.37 (-0.76, 0.02) 0.16 | 0.78 |

BMI: body mass index; TLM: total lean mass; ALM: appendicular lean mass; BMC: bone mineral content; TOF: total fat; TRF: trunk fat.

Subgroup analyses adjust for: age, race, eGFR, ratio of income-poverty, and education level.

**Table S2 : Subgroup analyses about age between urinary nickel and body mass components in NHANES 2017–2018.**

| **Outcomes** | **Age＜=31** | **Age＞31** | **P for interaction** |
| --- | --- | --- | --- |
|  | **β (95%CI) P-value** | **β (95%CI) P-value** |  |
| Weight, kg | -1.78 (-3.72, 0.17) 0.17 | 1.25 (-0.12, 2.62) 0.17 | 0.02 |
| BMI, kg/m2 | -0.32 (-0.82, 0.19) 0.31 | 0.45 (-0.05, 0.94) 0.17 | 0.05 |
| TLM, g/kg BM | -1.79 (-7.42, 3.84) 0.58 | -3.62 (-9.28, 2.04) 0.30 | 0.63 |
| ALM, g/kg BM | 2.80 (-0.40, 6.01) 0.19 | -2.96 (-9.27, 3.35) 0.43 | 0.13 |
| TRF, g/kg BM | 0.96 (-2.45, 4.36) 0.62 | 2.75 (-1.01, 6.51) 0.25 | 0.48 |
| TOF, g/kg BM | 3.00 (-3.20, 9.21) 0.41 | 3.62 (-2.29, 9.52) 0.32 | 0.89 |
| BMC, g/kg BM | -0.36 (-0.82, 0.09) 0.21 | -0.56 (-1.18, 0.05) 0.17 | 0.68 |

BMI: body mass index; TLM: total lean mass; ALM: appendicular lean mass; BMC: bone mineral content; TOF: total fat; TRF: trunk fat.

Subgroup analyses adjust for: gender, race, eGFR, ratio of income-poverty, and education level.

**Table S3: Subgroup analyses about race between urinary nickel and body mass components in NHANES 2017–2018.**

| **Outcomes** | **Race= Other Hispanic** | **Race = Non-Hispanic White** | **Race = Non-Hispanic Black** | **Race = Other Race** | **P for interaction** | |
| --- | --- | --- | --- | --- | --- | --- |
|  | **β (95%CI) P-value** | | | |  |  |
| Weight, kg | -1.90 (-3.99, 0.20) 0.33 | -0.66 (-2.96, 1.64) 0.67 | -0.55 (-2.09, 0.99) 0.61 | -0.12 (-3.51, 3.28) 0.96 | 0.79 |  |
| BMI, kg/m2 | -0.50 (-1.05, 0.05) 0.33 | -0.20 (-0.93, 0.53) 0.69 | 0.10 (-0.40, 0.60) 0.76 | -0.11 (-1.00, 0.77) 0.84 | 0.60 |  |
| TLM, g/kg BM | 1.32 (-4.71, 7.36) 0.74 | -5.43 (-13.92, 3.06) 0.43 | -2.55 (-9.18, 4.07) 0.59 | -2.68 (-14.17, 8.80) 0.73 | 0.73 |  |
| ALM, g/kg BM | 0.57 (-4.09, 5.23) 0.85 | -6.99 (-12.66, -1.32) 0.25 | 2.51 (-4.05, 9.06) 0.59 | -1.91 (-4.99, 1.16) 0.44 | 0.04 |  |
| TRF, g/kg BM | -2.44 (-5.22, 0.35) 0.34 | 2.32 (-2.78, 7.42) 0.54 | 2.23 (-1.91, 6.37) 0.48 | 2.35 (-4.53, 9.23) 0.62 | 0.27 |  |
| TOF, g/kg BM | -1.49 (-7.77, 4.79) 0.72 | 6.24 (-2.01, 14.49) 0.38 | 2.68 (-4.34, 9.70) 0.59 | 6.15 (-7.27, 19.57) 0.53 | 0.55 |  |
| BMC, g/kg BM | -0.10 (-0.61, 0.41) 0.76 | -0.30 (-1.18, 0.59) 0.63 | -0.55 (-1.15, 0.06) 0.33 | -0.36 (-1.31, 0.58) 0.59 | 0.65 |  |

BMI: body mass index; TLM: total lean mass; ALM: appendicular lean mass; BMC: bone mineral content; TOF: total fat; TRF: trunk fat.

Subgroup analyses adjust for: age, gender, eGFR, ratio of income-poverty, and education level.

**Table S4: Subgroup analyses about eGFR between urinary nickel and body mass components in NHANES 2017–2018.**

| **Outcomes** | **eGFR (CKD-EPI) <= 60** | **eGFR (CKD-EPI) > 60** | **P for interaction** |
| --- | --- | --- | --- |
|  | **β (95%CI) P-value** | **β (95%CI) P-value** |  |
| Weight | -0.89 (-3.58, 1.80) 0.56 | -0.57 (-1.74, 0.61) 0.42 | 0.82 |
| BMI | -0.35 (-1.34, 0.64) 0.54 | -0.01 (-0.36, 0.33) 0.94 | 0.51 |
| TLM | 14.67 (-16.81, 46.14) 0.43 | -2.58 (-7.11, 1.96) 0.35 | 0.31 |
| ALM | -3.06 (-13.36, 7.23) 0.60 | 0.37 (-2.80, 3.54) 0.83 | 0.53 |
| TRF | 10.25 (-10.13, 30.62) 0.40 | 1.56 (-1.08, 4.20) 0.33 | 0.42 |
| TOF | -16.40 (-51.22, 18.42) 0.42 | 3.29 (-1.37, 7.96) 0.26 | 0.29 |
| BMC | 1.22 (-2.84, 5.27) 0.60 | -0.44 (-0.73, -0.15) 0.06 | 0.43 |

BMI: body mass index; TLM: total lean mass; ALM: appendicular lean mass; BMC: bone mineral content; TOF: total fat; TRF: trunk fat.

Subgroup analyses adjust for: age, gender, race, ratio of income-poverty, and education level.

**Table S5: Subgroup analyses about ratio of income-poverty between urinary nickel and body mass components in NHANES 2017–2018.**

| **Outcomes** | **Economic condition = Low** | **Economic condition = Middle** | **Economic condition = High** | **P for interaction** |
| --- | --- | --- | --- | --- |
|  | **β (95%CI) P-value** | | |  |
| Weight, kg | -1.21 (-3.56, 1.15) 0.42 | 0.17 (-1.93, 2.28) 0.89 | -1.10 (-2.83, 0.64) 0.34 | 0.67 |
| BMI, kg/m2 | -0.14 (-0.85, 0.56) 0.73 | 0.02 (-0.58, 0.62) 0.95 | -0.04 (-0.58, 0.50) 0.90 | 0.96 |
| TLM, g/kg BM | 0.01 (-6.43, 6.46) 1.0 | -5.17 (-13.13, 2.79) 0.33 | -0.98 (-6.39, 4.43) 0.76 | 0.54 |
| ALM, g/kg BM | 1.49 (-2.10, 5.08) 0.50 | -1.57 (-8.11, 4.96) 0.68 | 1.42 (-5.57, 8.42) 0.73 | 0.73 |
| TRF, g/kg BM | 3.09 (-1.42, 7.61) 0.31 | 2.37 (-3.48, 8.22) 0.51 | -0.80 (-5.33, 3.73) 0.76 | 0.39 |
| TOF, g/kg BM | 1.04 (-5.61, 7.68) 0.79 | 5.12 (-3.62, 13.87) 0.37 | 2.11 (-3.72, 7.94) 0.55 | 0.75 |
| BMC, g/kg BM | -0.39 (-1.03, 0.26) 0.36 | -0.33 (-0.95, 0.28) 0.40 | -0.53 (-1.21, 0.15) 0.27 | 0.93 |

BMI: body mass index; TLM: total lean mass; ALM: appendicular lean mass; BMC: bone mineral content; TOF: total fat; TRF: trunk fat.

Subgroup analyses adjust for: age, gender, race, eGFR, and ratio of income-poverty.

**Table S6: Subgroup analyses about education level between urinary nickel and body mass components in NHANES 2017–2018.**

| **Outcomes** | **Education level = Less than high school** | **Education level = High school or general educational development** | **Education level = Above high school** | **P for interaction** |
| --- | --- | --- | --- | --- |
|  | **β (95%CI) P-value** | | |  |
| Weight, kg | -3.44 (-6.44, -0.43) 0.15 | 0.71 (-1.25, 2.67) 0.55 | -0.69 (-2.03, 0.64) 0.41 | 0.04 |
| BMI, kg/m2 | -0.91 (-1.63, -0.18) 0.13 | 0.31 (-0.09, 0.71) 0.27 | -0.04 (-0.45, 0.38) 0.88 | <0.01 |
| TLM, g/kg BM | 9.26 (-3.62, 22.15) 0.29 | -6.42 (-14.12, 1.29) 0.24 | -2.12 (-7.28, 3.05) 0.51 | 0.18 |
| ALM, g/kg BM | 5.36 (-3.02, 13.75) 0.34 | 0.61 (-5.97, 7.20) 0.87 | -0.02 (-4.50, 4.46) 0.99 | 0.60 |
| TRF, g/kg BM | -6.54 (-12.68, -0.39) 0.17 | 4.48 (-0.13, 9.09) 0.20 | 1.48 (-1.78, 4.74) 0.47 | 0.05 |
| TOF, g/kg BM | -9.51 (-22.18, 3.16) 0.28 | 6.38 (-0.91, 13.68) 0.23 | 3.06 (-2.36, 8.49) 0.38 | 0.15 |
| BMC, g/kg BM | 0.20 (-0.50, 0.90) 0.63 | -0.49 (-1.20, 0.21) 0.30 | -0.44 (-0.76, -0.12) 0.11 | 0.30 |

BMI: body mass index; TLM: total lean mass; ALM: appendicular lean mass; BMC: bone mineral content; TOF: total fat; TRF: trunk fat.

Subgroup analyses adjust for: age, gender, race, eGFR, and education level.
